# Supplementary material for: OpenPBTA: The Open Pediatric Brain Tumor Atlas
Source: Cell Genom. 2023 May 31;3(7):100340. doi: 10.1016/j.xgen.2023.100340 (PMC10363844; doi:10.1016/j.xgen.2023.100340)
Supplement: Document S1. Figures S1–S7 [file mmc1.pdf]

Cell Genomics, Volume *3*

Supplemental information

OpenPBTA: The Open Pediatric Brain Tumor Atlas

Joshua A. Shapiro, Krutika S. Gaonkar, Stephanie J. Spielman, Candace L. Savonen, Chante J. Bethell, Run Jin, Komal S. Rathi, Yuankun Zhu, Laura E. Egolf, Bailey K. Farrow, Daniel P. Miller, Yang Yang, Tejaswi Koganti, Nighat Noureen, Mateusz P. Koptyra, Nhat Duong, Mariarita Santi, Jung Kim, Shannon Robins, Phillip B. Storm, Stephen C. Mack, Jena V. Lilly, Hongbo M. Xie, Payal Jain, Pichai Raman, Brian R. Rood, Rishi R. Lulla, Javad Nazarian, Adam A. Kraya, Zalman Vaksman, Allison P. Heath, Cassie Kline, Laura Scolaro, Angela N. Viaene, Xiaoyan Huang, Gregory P. Way, Steven M. Foltz, Bo Zhang, Anna R. Poetsch, Sabine Mueller, Brian M. Ennis, Michael Prados, Sharon J. Diskin, Siyuan Zheng, Yiran Guo, Shrivats Kannan, Angela J. Waanders, Ashley S. Margol, Meen Chul Kim, Derek Hanson, Nicholas Van Kuren, Jessica Wong, Rebecca S. Kaufman, Noel Coleman, Christopher Blackden, Kristina A. Cole, Jennifer L. Mason, Peter J. Madsen, Carl J. Koschmann, Douglas R. Stewart, Eric Wafula, Miguel A. Brown, Adam C. Resnick, Casey S. Greene, Jo Lynne Rokita, Jaclyn N. Taroni, Children's Brain Tumor Network, and Pacific Pediatric Neuro-Oncology Consortium

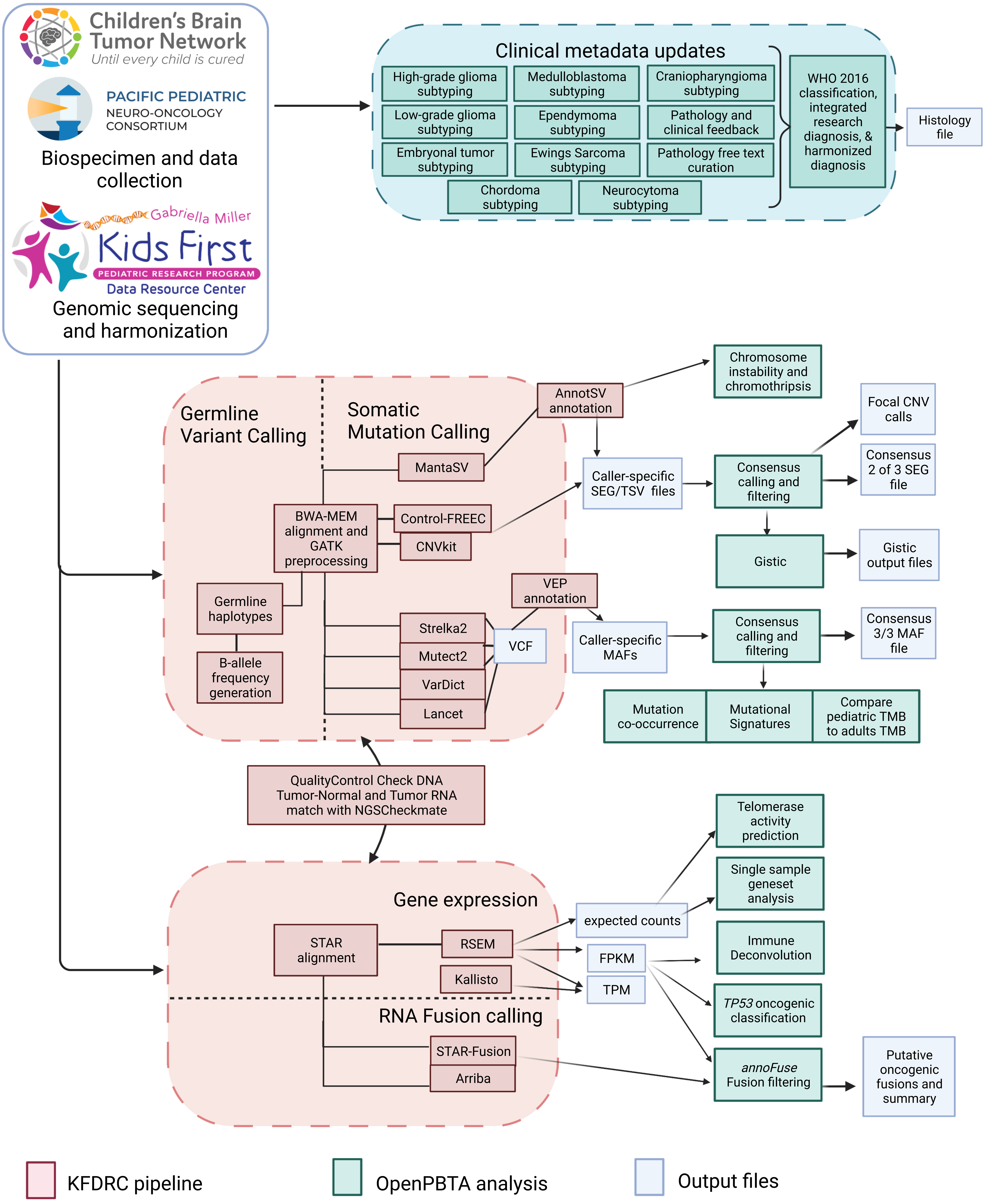

Figure S1. OpenPBTA Project Workflow, Related to Figure 1. Biospecimens and data were collected by CBTN and PNOC. Genomic sequencing and harmonization (orange boxes) were performed by the Kids First Data Resource Center (KFDRC). Analyses in the green boxes were performed by contributors of the OpenPBTA project. Output files are denoted in blue. Figure created with BioRender.com.

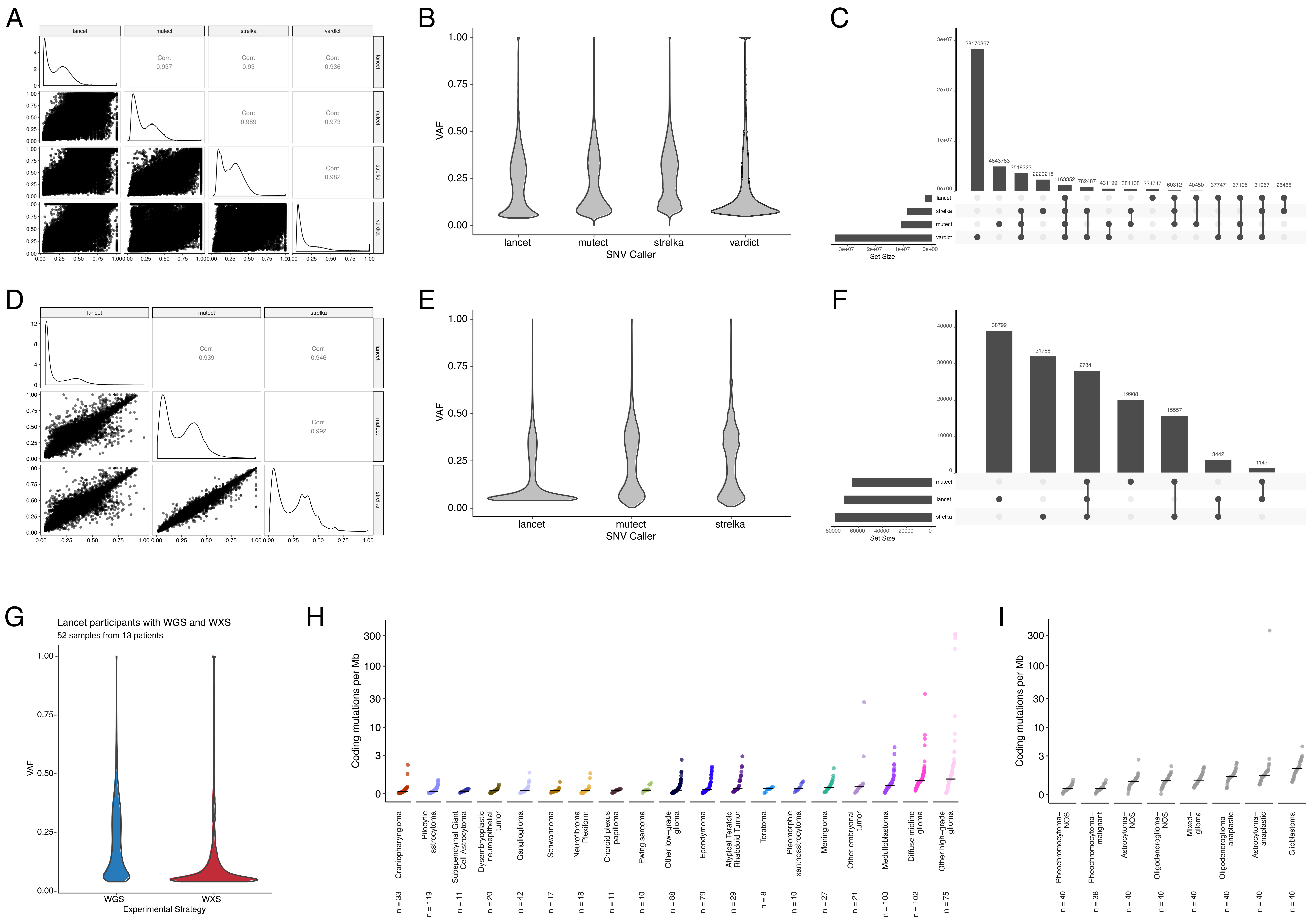

Figure S2. Validation of Consensus SNV calls and Tumor Mutation Burden, Related to Figures 2 and 3. Correlation (A) and violin (B) plots of mutation variant allele frequencies (VAFs) comparing the variant callers (Lancet, Strelka2, Mutect2, and VarDict) used for PBTA samples. UpSet plot (C) showing overlap of variant calls. Correlation (D) and violin (E) plots of mutation variant allele frequencies (VAFs) comparing the variant callers (Lancet, Strelka2, and Mutect2) used for TCGA samples. UpSet plot (F) showing overlap of variant calls. Violin plots (G) showing VAFs for Lancet calls performed on WGS and WXS from the same tumor (n = 52 samples from 13 patients). Cumulative distribution TMB plots for PBTA (H) and TCGA (I) tumors using consensus SNV calls.

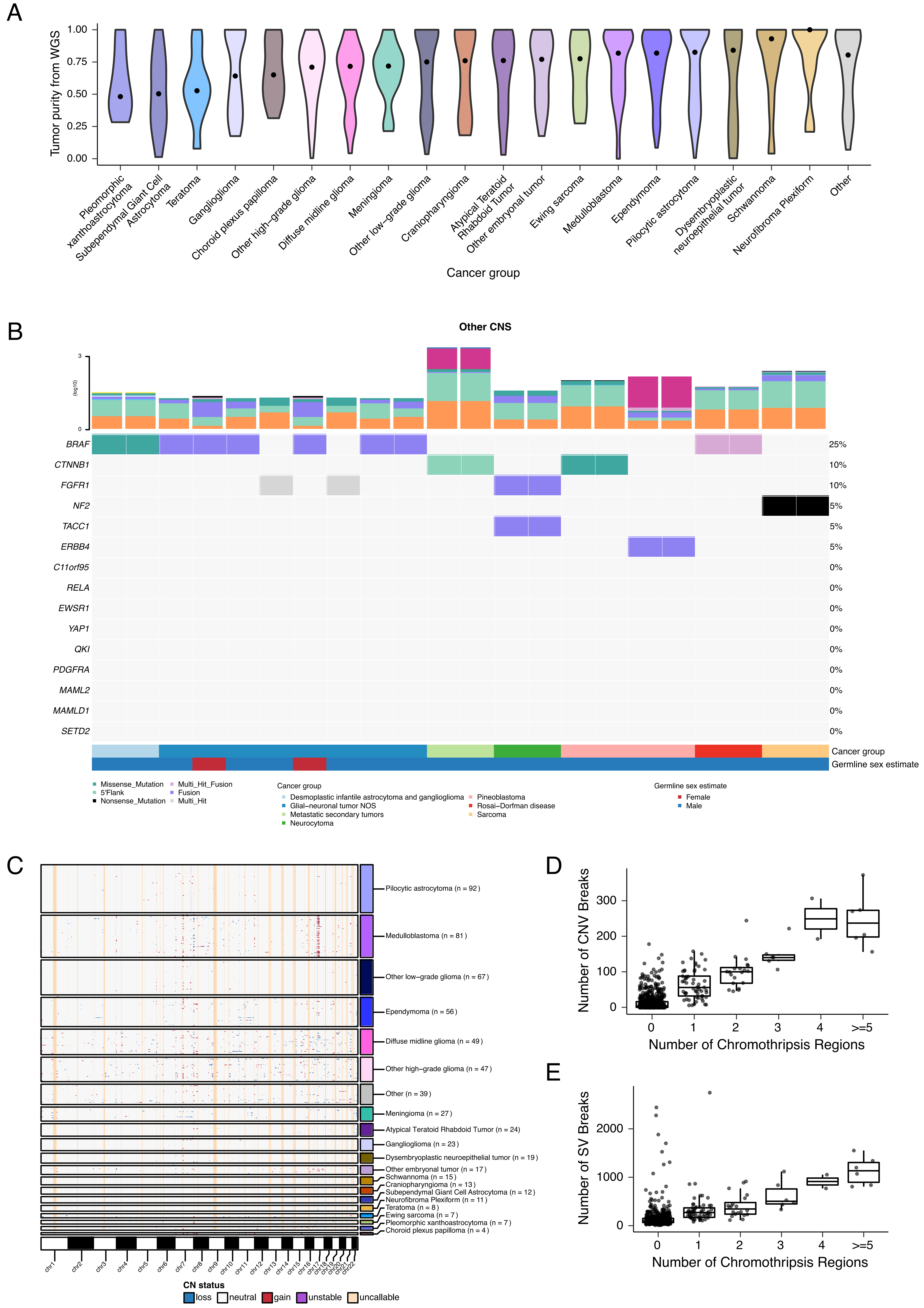

Figure S3. Genomic instability of pediatric brain tumors, Related to Figures 2 and 3. (A) Violin plots of tumor purity by cancer group. Dots represent the group median. (B) Oncoprint of canonical somatic gene mutations, CNVs, fusions, and TMB (top bar plot) for the top mutated genes across rare CNS tumors: desmoplastic infantile astrocytoma and ganglioglioma (n = 2), germinoma (n = 4), glial-neuronal NOS (n = 8), metastatic secondary tumors (n = 2), neurocytoma (n = 2), pineoblastoma (n = 4), Rosai-Dorfman disease (n = 2), and sarcomas (n = 4). Patient sex (`Germline sex estimate`) and tumor histology (`Cancer Group`) are displayed as annotations at the bottom of each plot. Multiple CNVs are denoted as a complex event. The n denotes the number of unique tumors with one tumor per patient used. (C) Genome-wide plot of CNV alterations by broad histology. Each row represents one sample. Box and whisker plots of number of CNV breaks (D) or SV breaks (E) by number of chromothripsis regions. Box plot represents 5% (lower whisker), 25% (lower box), 50% (median), 75% (upper box), and 95% (upper whisker) quantiles.

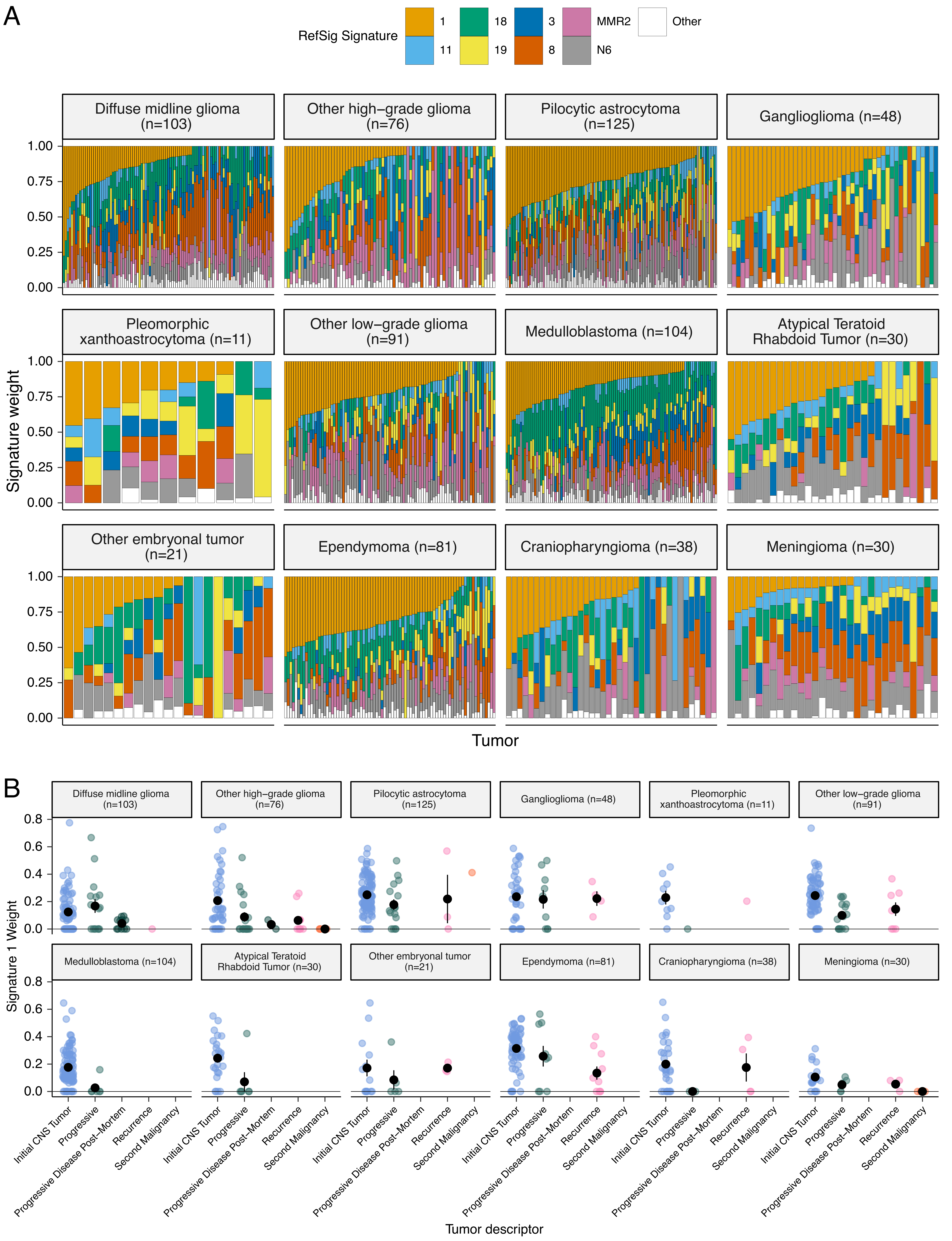

Figure S4. Mutational signatures in pediatric brain tumors, Related to Figure 3. (A) Sample-specific RefSig signature weights across cancer groups ordered by decreasing Signature 1 exposure. (B) Proportion of Signature 1 plotted by phase of therapy for each cancer group.

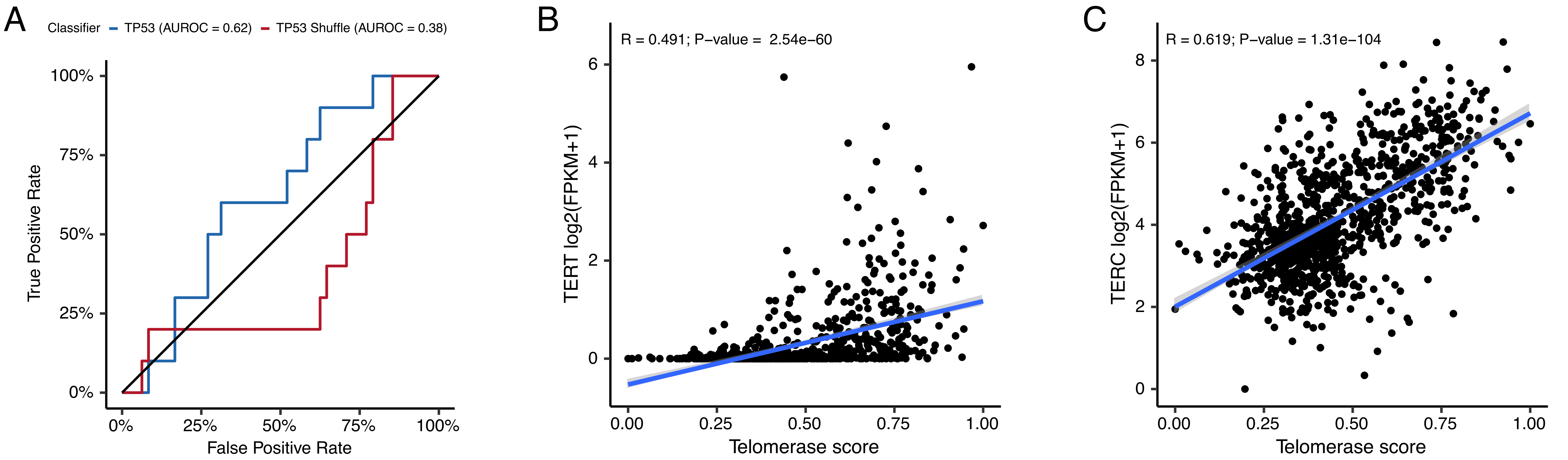

Figure S5. Quality control metrics for *TP53* and EXTEND scores, Related to Figure 4. (A) Receiver Operating Characteristic for _TP53_ classifier run on FPKM of poly-A RNA-Seq samples. Correlation plots for telomerase scores (EXTEND) with RNA expression of *TERT* (B) and *TERC* (C). Red dots in B and C denote samples with known *TERT* promoter (TERTp) mutations.

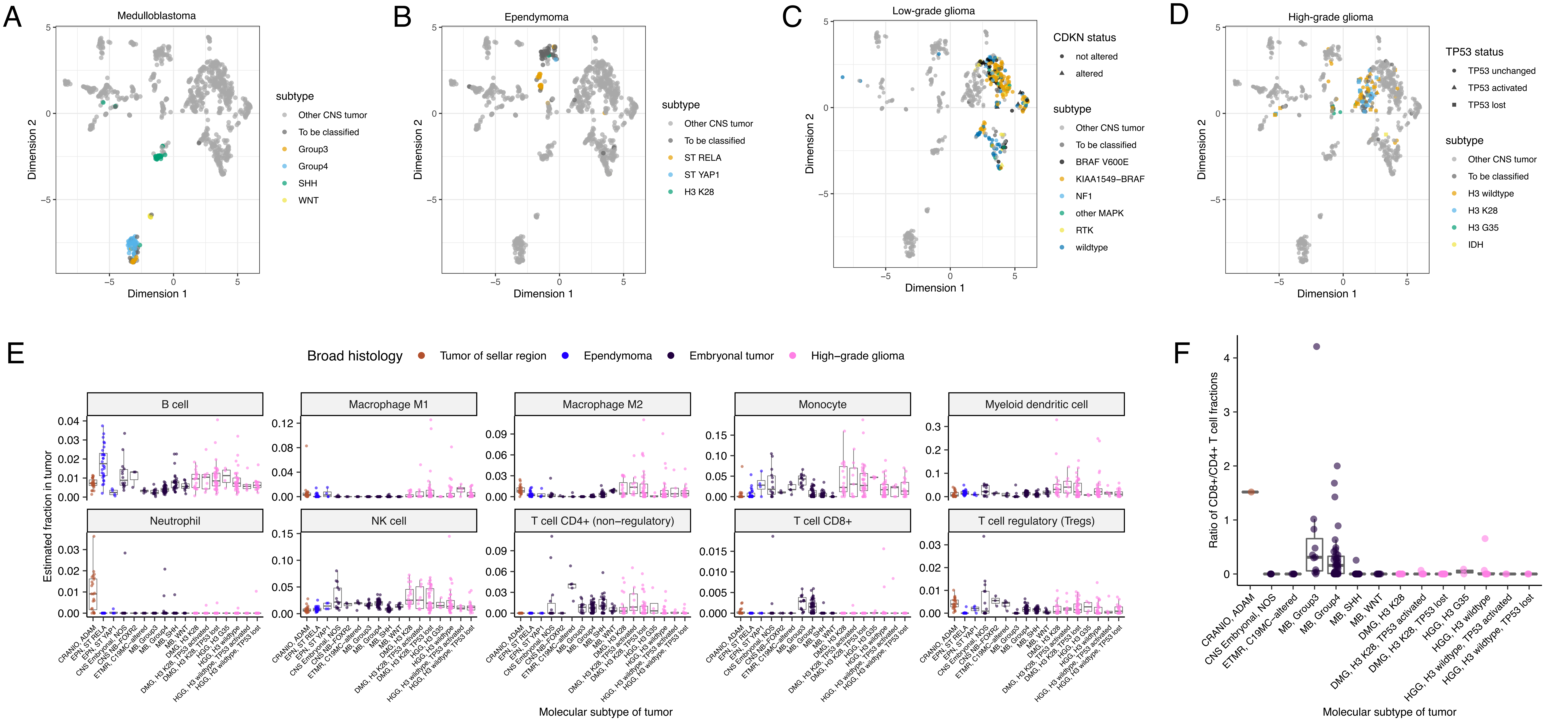

Figure S6. Subtype-specific clustering and immune cell fractions, Related to Figure 5. First two dimensions from UMAP of sample transcriptome data with points colored by `molecular_subtype` for medulloblastoma (A), ependymoma (B), low-grade glioma (C), and high-grade glioma (D). (E) Box plots of quanTIseq estimates of immune cell fractions in histologies with more than one molecular subtype with n >=3. (F) Box plots of the ratio of immune cell fractions of CD8+ to CD4+ T cells in histologies with more than one molecular subtype with n >=3. Box plot represents 5% (lower whisker), 25% (lower box), 50% (median), 75% (upper box), and 95% (upper whisker) quantiles.

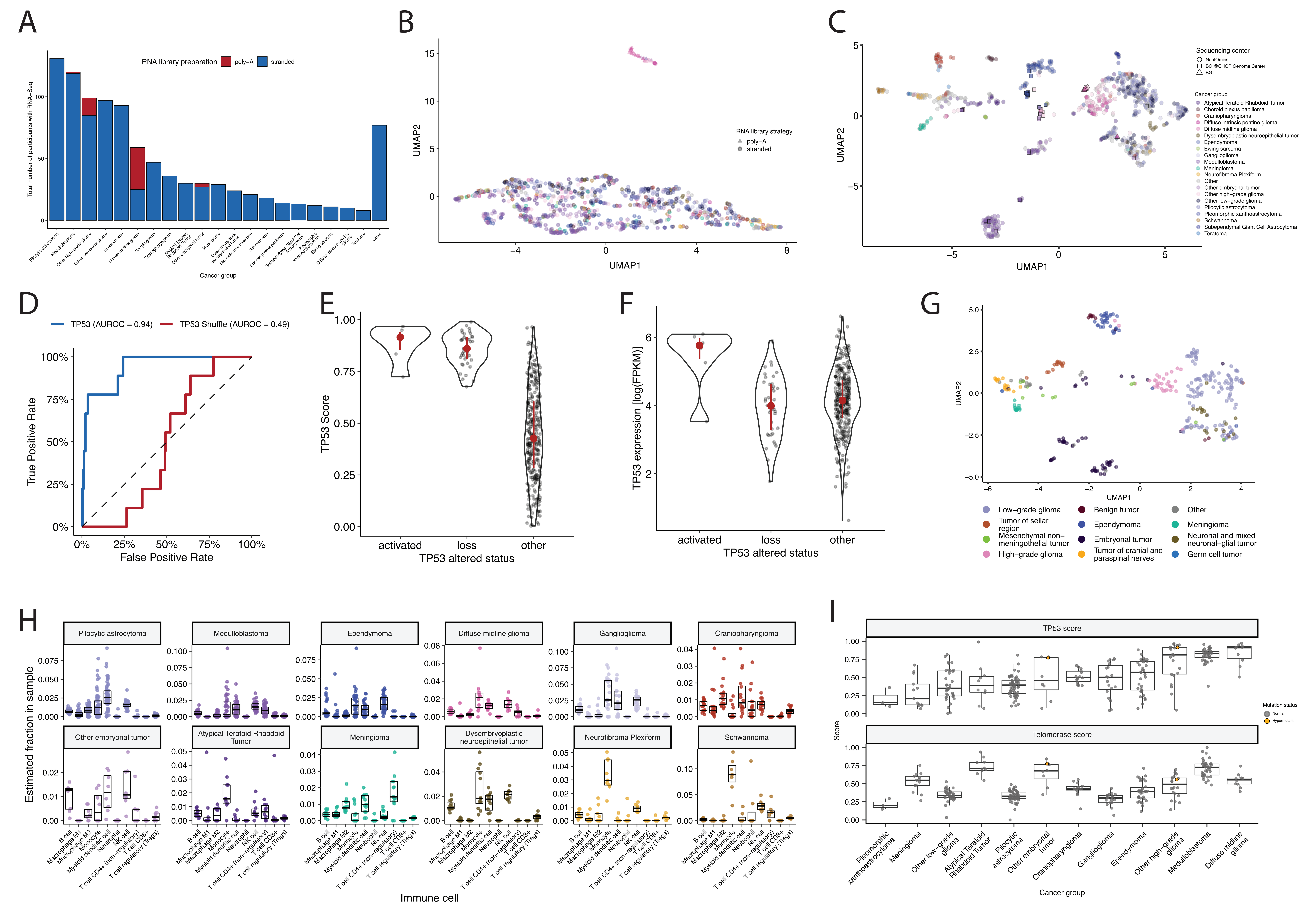

Figure S7. RNA batch and tumor purity assessment, Related to Figures 4 and 5. Bar plot (A) and UMAP (B) of RNA-Seq samples by cancer group and library preparation method. (C) UMAP of RNA-Seq samples by cancer group and sequencing center. For (D-I), RNA-Seq samples were thresholded by median cancer group tumor purity and transcriptomic analyses in **Figure 4A-D** (D-G) and **Figure 5A,C** (H-I) were repeated.
